# Screening and validation of differentially expressed genes in adipose tissue of patients with obesity and type 2 diabetes mellitus

**DOI:** 10.17305/bb.2023.9498

**Published:** 2024-02-01

**Authors:** Xuewei Tong, Chunyan Liu, Mengjie Liang, Xueyan Ye, Zhaohui Deng, Xin Zhang

**Affiliations:** 1 Department of Clinical Laboratory, Hospital of Xinjiang Production and Construction Corps/Second Affiliated Hospital, Medical School of Shihezi University, Urumqi, Xinjiang, China; 2 Clinical Laboratory Center, People’s Hospital of Xinjiang Uygur Autonomous Region, Urumqi, Xinjiang, China; 3 Prenatal Diagnosis Center, Urumqi Maternal and Child Health Hospital, Urumqi, Xinjiang, China

**Keywords:** Type 2 diabetes mellitus (T2DM), obesity, white adipose tissue (WAT), bioinformatics, differentially expressed genes (DEGs)

## Abstract

White adipose tissue (WAT) plays a pivotal role in the onset of type 2 diabetes mellitus (T2DM) and obesity. Despite its significance the underlying pathogenesis and key genes associated with it remain elusive. In our study, we screened the differentially expressed genes (DEGs) in intra-abdominal WAT of T2DM patients with obesity, as well as those with simple obesity, aiming to lay a foundational theory for an in-depth investigation of T2DM pathogenesis and the identification of novel therapeutic targets. Gene expression datasets (GSE16415 and GSE71416) were retrieved from the Gene Expression Omnibus (GEO) database. We employed R for screening DEGs and conducted a functional enrichment analysis using the Metascape database. Combined Lasso regression and Boruta feature selection algorithms were used to identify key DEGs. Subsequently, these were cross-verified using the GSE29231 dataset. Samples and medical records were collected from clinical study participants. The mRNA and protein expressions of the key DEGs were verified using quantitative reverse transcription polymerase chain reaction and western blotting, respectively. We discerned a total of 130 DEGs, with 40 being upregulated and 90 downregulated. Functional and pathway enrichment analyses illuminated that these genes are instrumental in mediating metabolite and energy production, neutrophil-mediated immunity, and other associated biological processes. This includes their involvement in the tricarboxylic acid cycle, glycolysis/gluconeogenesis, peroxisome proliferator-activated receptors, and other signaling pathways. Two genes, *CIDEA* and *FSCN1* emerged as key DEGs. The low expression of *CIDEA* and high expression of *FSCN1* in the T2DM and obesity groups were verified in clinical samples (*P* < 0.05). We established that *CIDEA* and *FSCN1* manifest significant differential expression in T2DM patients who are obese. This suggests their potential as risk assessment markers and therapeutic targets for T2DM.

## Introduction

Type 2 diabetes mellitus (T2DM) is among the most prevalent chronic metabolic diseases globally, with its incidence rising each year. Due to its detrimental impact on quality of life and the significant societal burden it imposes, T2DM merits increased attention [1–3]. Obesity stands as a pivotal risk factor for the onset of prediabetes and T2DM [4, 5]. The pathogenesis of both T2DM and obesity might be linked to alterations in the function of white adipose tissue (WAT). Notably, dysfunctional WAT in obese individuals can heighten the risk of insulin resistance (IR) and subsequently, T2DM [6, 7]. However, it’s crucial to note that not all obese individuals develop diabetes, and there are instances where those with elevated body mass index (BMI) display normal insulin sensitivity and glucolipid metabolic phenotypes [8, 9]. Consequently, the mere presence of an increased amount of WAT might not sufficiently account for its functional health. This underscores the need for more in-depth studies to thoroughly grasp the pathological roles and mechanisms of obesity and IR in T2DM’s progression.

In recent years, the advent and application of high-throughput sequencing technology have led to the generation of vast amounts of gene expression profile data [10]. Through applied bioinformatics, key differentially expressed genes (DEGs) and biomarkers of T2DM can be screened by analyzing the mRNA expression in the WAT of patients with both obesity and T2DM, as well as those with simple obesity. This analysis, derived from the sequencing database, could offer a more intuitive perspective, highlighting the connections between obesity and T2DM [11, 12].

In this study, we investigated the gene expression patterns and pathways associated with T2DM in the context of obesity. We employed bioinformatics-based analyses to screen the key DEGs in the intra-abdominal omentum WAT, using gene expression profile data from two groups: T2DM patients with obesity and those with simple obesity, sourced from the Gene Expression Omnibus (GEO). This data was then validated both at the molecular and protein levels. Our findings lay the groundwork for a deeper understanding of the pathophysiology of T2DM in conjunction with obesity and contribute to the discovery of novel therapeutic targets for its treatment.

## Materials and methods

### Screening and correction of data

**Figure S1.**
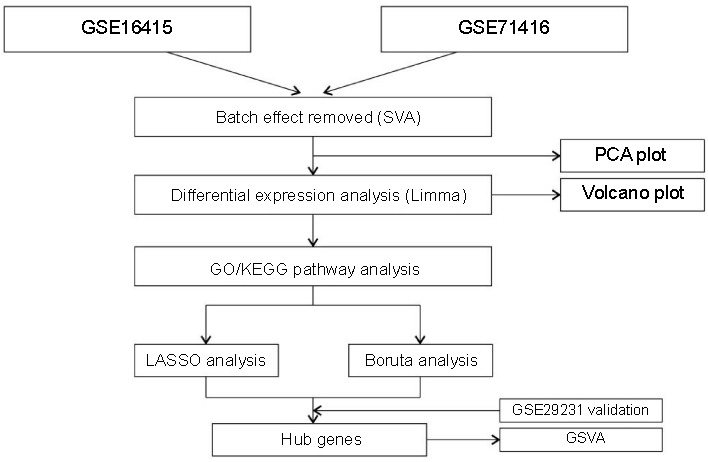
**Screening process of key differentially expressed genes (DEGs).** SVA: Surrogate variable analysis; PCA: Principal component analysis; GSVA: Gene set variation analysis; GO: Gene ontology; KEGG: Kyoto Encyclopedia of Genes and Genomes.

Gene expression data related to intra-abdominal WAT in both T2DM groups (accession numbers GSE16415 and GSE71416) were downloaded from the National Centre for Bioinformatics GEO database (https://www.ncbi.nlm.nih.gov/gds). Specific information from all datasets can be found in Table S1. The screening process for key DEGs in this study is shown in Figure S1.

### Data pre-processing and identification of DEGs

In this study, data batch correction was conducted in the R statistical environment using the ComBat function from the surrogate variable analysis (sva) package for data merging, normalization, and expression value calculation for known batch effects. The analysis and screening of DEGs between sample groups were carried out using the limma package. A difference multiplier of |logFC| >1 was set for differential gene expression based on the data’s characteristics, and a corrected *P* value < 0.05 was considered statistically significant. The size and significance of the difference in expression for each gene between the two groups were analyzed.

### Enrichment analysis of DEGs

Gene Ontology (GO) and Kyoto Encyclopedia of Genes and Genomes (KEGG) pathway analyses were performed on the DEGs using the Metascape database. A minimum overlap of ≥3 and *P* < 0.05 were considered statistically significant.

### Identification of key DEGs

In the R statistical environment, we employed the “glmnet” package for Lasso regression analysis and the “Boruta” package for the Boruta algorithm. We combined the common Lasso regression and the Boruta algorithm DEGs. The T2DM-related datasets and corresponding clinical data in GSE29231 were downloaded from the GEO database. A differential analysis of the screened DEGs was performed to identify the key DEGs. Statistical significance was set at *P* < 0.05.

### Gene set variation analysis of key DEGs

The gene set was downloaded from the Molecular Signatures Database (v7.0). The gene set variation analysis (GSVA) algorithm was used to comprehensively score the gene set and evaluate the crucial signaling pathways associated with key DEGs in T2DM.

### Expression validation of DEGs

#### Selection of study participants and data collection

The selected patients underwent elective abdominal surgery in the Xinjiang Production and Construction Corps Hospital between April 2020 and February 2021. This included eight patients in the normal control group (Normal Control [NC]), 12 in the simple obesity group (Obese [OB]), and four in the T2DM and obesity group (T2DM+OB). General data, such as sex, age, and medical history of the study participants, were collected. Height and weight measurements were taken after fasting, crown-free, and without shoes. BMI was calculated using the formula: Weight (kg)/[height (m)]^2^.

The inclusion criteria for this study were as follows: Diagnosis of T2DM according to the 1999 World Health Organization definition (typical symptoms accompanied by a fasting blood glucose level of ≥7.0 mmol/L or a postprandial blood glucose level of ≥ 11.1 mmol/L) [13]; Diagnosis of obesity according to the “Guidelines for the Prevention and Control of Overweight and Obesity in Chinese Adults” criteria: A BMI of 18.5 kg/m^2^ to <24 kg/m^2^ is considered normal, while a BMI of ≥28 kg/m^2^ is classified as obese [14]; Age >18 years; For patients diagnosed with T2DM, no prior treatments for diabetes, including diet and exercise therapy; Absence of a history of weight loss drug usage within the past six months; Informed consent to participate in the study.

The exclusion criteria were as follows: Patients with other combined forms of diabetes and obesity, such as type 1 diabetes, pheochromocytoma, cortisolism, or secondary obesity; Patients with disorders, such as severe cardiopulmonary insufficiency, thyroid dysfunction, and advanced malignancy; Patients with severe infections in the urinary, respiratory, and digestive systems; Pregnant women.

#### Adipose tissue sample collection

Approximately 100 mg of intra-abdominal WAT was collected from the greater omentum of the study participants. This material was collected within 30 min following adipose tissue excision (ex vivo), quick-frozen in liquid nitrogen, and then stored at −80 ^∘^C for subsequent experimental index analysis.

#### SYBR green quantitative reverse transcription polymerase chain reaction

Total RNA from WAT was extracted using the Trizol method (EMD Millipore, Burlington, MA, USA). The concentration and purity of the RNA were detected using a Nanodrop 2000 nucleic acid detector (Thermo Fisher Scientific, Waltham, MA, USA). Reverse transcription reactions were carried out using the All-Style Gold EasyScript^®^ One-Step gDNA Removal and cDNA Synthesis SuperMix. Quantitative reverse transcription polymerase chain reaction (qRT-PCR) assays were performed using the All-Style Gold PerfectStart^TM^ Green qPCR SuperMix. PCR conditions involved 45 cycles of: 94 ^∘^C for 30 s, 94 ^∘^C for 5 s, 60 ^∘^C for 15 s, and 72 ^∘^C for 10 s. All analyses were carried out in triplicate, using the β-actin housekeeping gene as an internal reference. Relative mRNA expression levels were determined by calculating the ΔCT and 2^−ΔCT^ values with the mean CT of the target gene normalized to the internal reference gene. The primer sequences utilized for these experiments can be found in Table S2.

#### Western blotting

Total protein was extracted from 30 to 50 mg of adipose tissue, and protein concentrations were determined for all samples. The supernatant was separated using 12% sodium dodecyl–sulphate polyacrylamide gel electrophoresis and transferred to nitrocellulose membranes. These membranes were placed in a flat dish containing a 5% blocking solution and blocked at room temperature for two hours. After that, the nitrocellulose membranes were incubated in a 5% blocking solution containing primary antibodies against the target proteins and left overnight in a refrigerator at 4 ^∘^C. After washing with 1× Tris-buffered saline (TBS) containing Tween 20, the washed nitrocellulose membranes were transferred to 5% TBS containing the secondary antibody and incubated in a shaker at room temperature for two hours. Following enhanced chemiluminescence development and fixation, X-ray films were rinsed with distilled water and dried at room temperature. The X-ray films were scanned, and grayscale values of the target and internal reference bands as well as their ratios were analyzed using ImageJ image processing software (NIH, Bethesda, MD, USA).

### Ethical statement

This study was approved by the Ethics Committee of Xinjiang Production and Construction Corps Hospital (approval number: 20200301). All study procedures were in compliance with the ethical standards of the relevant national and institutional committees on human experimentation as well as the Declaration of Helsinki of 1975, as revised in 2008. Written informed consent was obtained from all individual participants included in the study.

### Statistical analysis

Bioinformatics analyses were performed in the R statistical environment (version 3.6; R Foundation for Statistical Computing, Vienna, Austria). Statistical analyses were carried out using SPSS 25.0 (IBM, Armonk, NY, USA) and Excel 2016 (Microsoft, Redmond, WA, USA) software. Non-normally distributed data are expressed as medians (interquartile spacing), and non-parametric tests (Mann–Whitney *U* tests) were used for comparisons between groups. Count data were expressed as the number of cases (percentage), and χ^2^ tests (chi-square tests) were used for comparisons between groups, with *P* < 0.05 indicating a statistically significant difference.

## Results

### Data normalization and DEG screening

**Figure 1.**
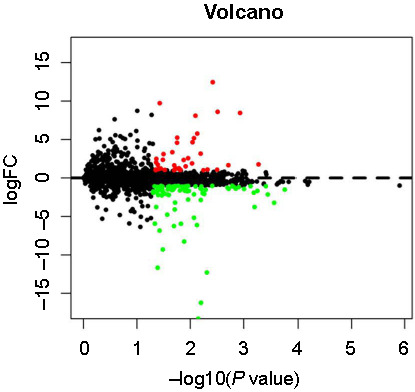
Volcano plot depicting the differentially expressed genes.

**Figure S2.**
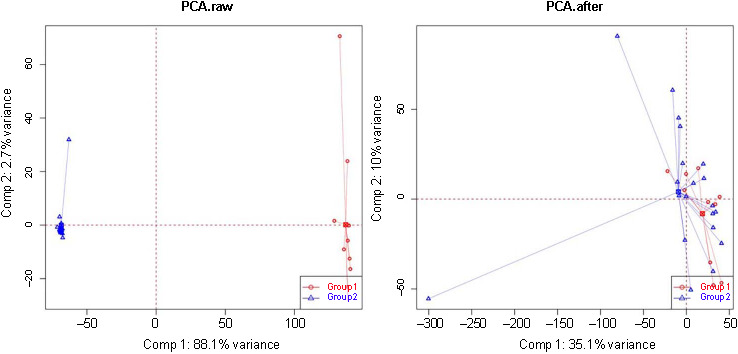
**Two-dimensional principal component analysis cluster diagram before/after sample calibration.** PCA: Principal component analysis.

Batch effects in the microarray data were corrected for using the sva package, and the differences before and after correction were demonstrated by principal component analysis plots, as shown in Figure S2. We performed differential gene identification on the standardized data, identifying 130 DEGs between T2DM patients with obesity and those with simple obesity. This includes 40 upregulated and 90 downregulated genes. The DEGs are visualized in a volcano plot: upregulated genes are marked in red, downregulated genes in green, and genes with no statistical significance in black (Figure 1). Detailed gene information can be found in Table S3.

### GO functional enrichment and KEGG pathway enrichment analysis

**Figure 2.**
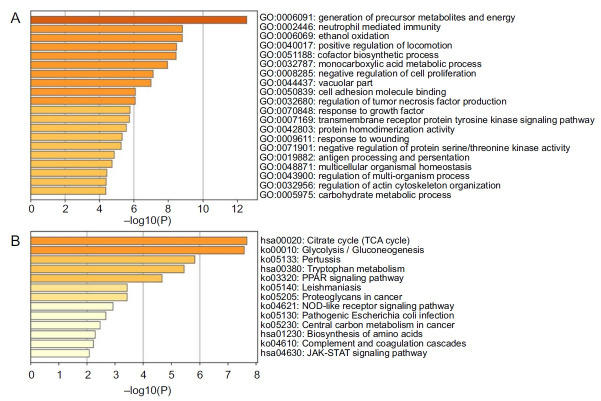
(A) Gene Ontology enrichment analysis of the differentially expressed genes (DEGs); (B) Kyoto Encyclopedia of Genes and Genomes enrichment analysis of the DEGs.

The GO enrichment analysis indicated that the DEGs were primarily associated with the generation of precursor metabolites and energy, neutrophil-mediated immunity, cofactor biosynthetic processes, and other biological processes such as the positive regulation of locomotion (Figure 2A). The KEGG enrichment analysis highlighted the enrichment in pathways including the tricarboxylic acid (TCA) cycle, glycolysis/gluconeogenesis, pertussis, tryptophan metabolism, and the PPAR signaling pathway (Figure 2B).

### Identification of key DEGs

**Figure 3.**
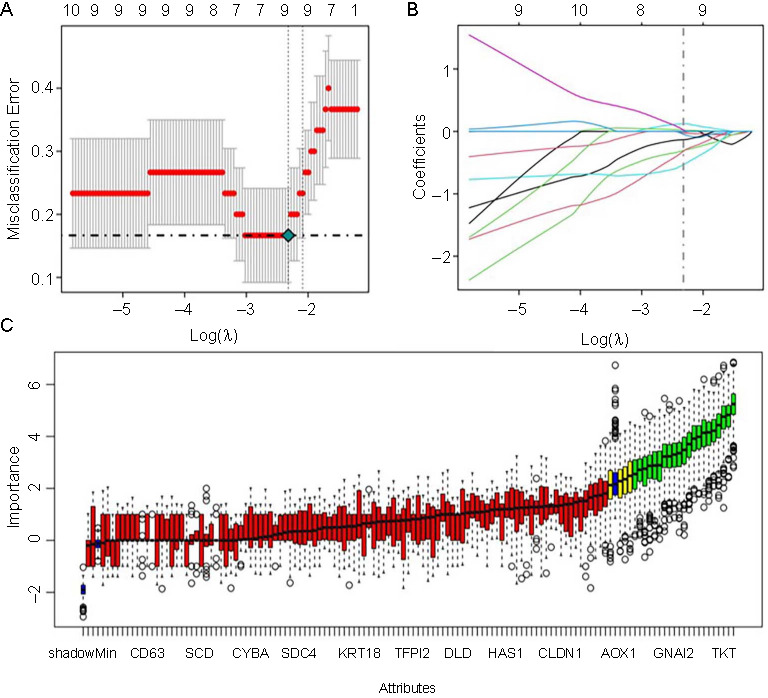
(A) Selection process of penalty parameters of fold cross-validation; (B) Lasso fitting curve; (C) Box plots of Boruta algorithm features.

Lasso regression models were developed using 130 genes from the initial screening results. This regression identified 10 key DEGs for the disease groups, as depicted in Figure 3A and 3B. Specific gene information is shown in Table S4. The Boruta algorithm identified 19 key DEGs in the disease group, as shown in Figure 3C. Specific gene information is shown in Table S5.

**Figure S3.**
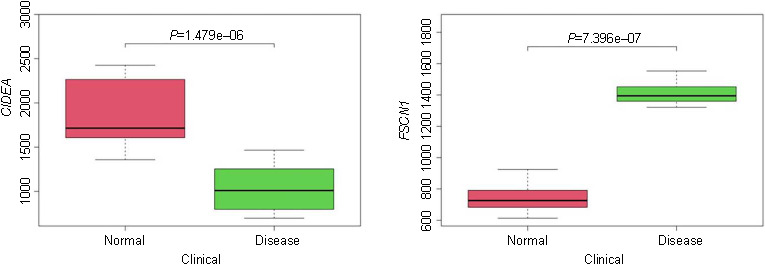
Analysis of differences in external datasets of *CIDEA* and *FSCN1*.

From the intersection of the Lasso regression and Boruta feature selection algorithms, six key DEGs were identified: *CIDEA*, *TKT*, *CGN*, *FSCN1*, *ECHDC3*, and *ANXA3*. Additionally, we downloaded an obese T2DM-related dataset from the GEO public database along with the corresponding clinical data from GSE29231 to analyze differences in these key DEGs. Our analysis revealed statistically significant differences in the expression of *CIDEA* (*P* ═ 1.479e-06) and *FSCN1* (*P* ═ 7.396e-07), as illustrated in Figure S3. Consequently, *CIDEA* and *FSCN1* emerged as the key DEGs in our study.

### GSVA analysis of key DEGs

**Figure 4.**
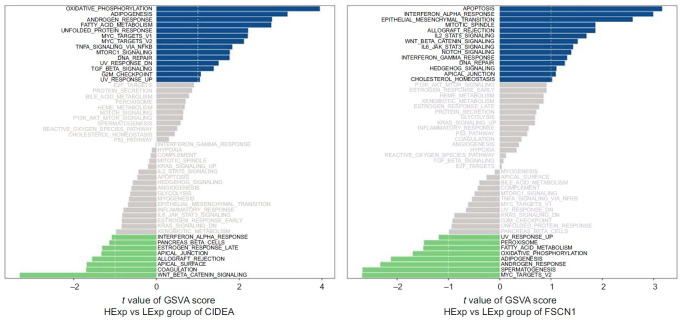
(A) Gene set variation analysis enrichment of *CIDEA*; (B) Gene set variation analysis enrichment of *FSCN1*.

The results from GSVA showed that the highly expressed gene *CIDEA* predominantly participates in metabolic-related signaling pathways, including oxidative phosphorylation, adipogenesis, and fatty acid metabolism. Conversely, the highly expressed *FSCN1* is mainly associated with inflammation-related signaling pathways, such as Wnt/β-linked protein, C3, interleukin-2, and transcriptional activator 5 signaling pathways (Figure 4).

### Comparison of the general characteristics of the study participants

Statistical analysis revealed that the weight and BMI of the OB and T2DM+OB groups were significantly higher (*P* < 0.05) than those of the NC group, as shown in Table 1.

**Table 1:** Participant characteristics

**Indicators**	**NC group**	**OB group**	**T2DM+OB group**	***χ^2^***	***P***
Number of cases (*n*)	8	12	4	–	–
Age (years)	49.00 [38.25, 59.00]	51 [43.75, 58.00]	57.50 [49.75, 63.00]	1.582	0.453
Height (cm)	168.5 [159.30, 176.00]	166 [160.5, 170.00]	160.00 [157.00, 162.25]	3.170	0.205
Body weight (kg)	60.00 [51.50, 65.75]	85 [80.75, 95.00]^*^	74.00 [73.00, 76.50]^▴#^	15.424	<0.001
BMI (kg/m^2^)	21.05 [20.34, 21.65]	31.05 [29.84, 33.85]^*^	29.26 [28.30, 30.06]^▴^	16.769	<0.001

Data are presented as median with interquartile range. *P < 0.05, NC vs OB; ^▴^*P* < 0.05, NC vs. T2DM+OB; ^#^*P* < 0.05, OB vs T2DM+OB. NC: Normal Control; OB: Obese; T2DM: Type 2 diabetes mellitus; BMI: Body mass index.

### qRT-PCR validation results

**Figure 5.**
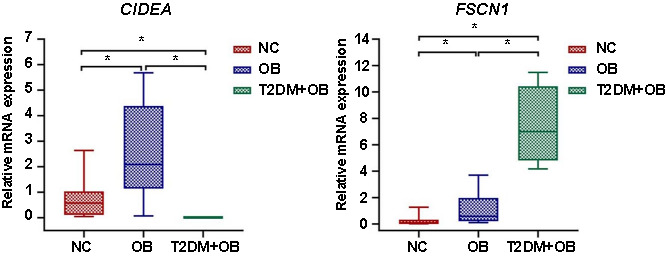
**(A) Relative mRNA expression levels of *CIDEA*; (B) Relative mRNA expression levels of *FSCN1*.** NC: Normal Control; OB: Obese; T2DM: Type 2 diabetes mellitus.

qRT-PCR analysis revealed that the mRNA expression level of *CIDEA* in the T2DM+OB group was significantly lower than those in the NC and OB groups (*P* < 0.05), and significantly higher in the OB group compared to the NC group (*P* < 0.05). The mRNA levels of *FSCN1* in the T2DM+OB group were significantly higher than those in the NC and OB groups (*P* < 0.05), and higher in the OB group compared to the NC group (*P* < 0.05), as shown in Figure 5.

### Western blot validation results

**Figure 6.**
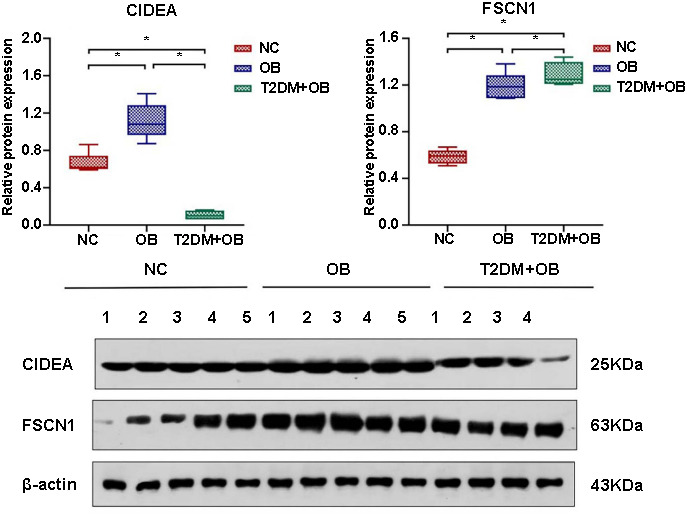
**(A) Relative protein expression levels of CIDEA; (B) Relative protein expression levels of FSCN1; (C) Original western blot images.** NC: Normal Control; OB: Obese; T2DM: Type 2 diabetes mellitus.

Western blot analysis suggested that the protein levels of CIDEA in the T2DM+OB group were significantly lower than those in the NC and OB groups (*P* < 0.05), and significantly higher in the OB group compared to the NC group (*P* < 0.05). The protein levels of FSCN1 in the T2DM+OB group were significantly higher than those in the NC and OB groups (*P* < 0.05), and significantly higher in the OB group compared to the NC group (*P* < 0.05). The mRNA and protein expression levels of CIDEA and FSCN1 within each group were consistent (Figure 6).

## Discussion

While obesity is widely recognized as a major risk factor for T2DM, studies indicate that some individuals with obesity still maintain a normal glucolipid metabolic phenotype. Although prior research has identified key DEGs in the WAT of obese individuals that potentially regulate T2DM development, the specific underlying mechanisms remain elusive [15, 16]. Therefore, a comprehensive analysis of the transcriptome of WAT in patients with T2DM and obesity and those with simple obesity is required. Such an investigation would deepen our understanding of the pathological mechanisms driving the simultaneous occurrence of obesity and T2DM, offering a more intuitive insight into their connection.

In this study, we analyzed high-throughput gene expression profile microarray data from intra-abdominal large omental WAT samples. These samples were collected from 10 individuals with simple obesity and 19 individuals with both T2DM and obesity, all sourced from the GEO database. Utilizing applied bioinformatics methods, we screened a total of 130 DEGs. KEGG enrichment analysis revealed that the DEGs are associated with the TCA cycle, glycolysis/gluconeogenesis, pertussis, tryptophan metabolism, PPAR signaling, and other inflammatory pathways. Therefore, the screened DEGs are involved in and regulate the pathophysiological mechanisms of WAT-induced IR and T2DM. The modulation of the adipose inflammatory response and its related signaling pathways by these DEGs offers a foundational basis for future research, potentially guiding strategies for the prevention and treatment of T2DM [17, 18].

We used both Lasso regression and the Boruta two-feature algorithms to establish the best classification model. We further identified *CIDEA* and *FSCN1* as the key DEGs of T2DM with obesity through validation using the GSE29231 dataset. The product encoded by *CIDEA*, known as DNA fragmentation factor α-like effector A, induces cell death and acts as an intra-organismal lipid droplet-associated protein. It enhances adipose tissue lipid storage capacity by increasing intracellular lipid droplet volume [19]. *CIDEA* is highly expressed in WAT and is positively correlated with a healthy metabolic phenotype in humans [20, 21]. Puri et al. (2008) reported that the protein expression level of CIDEA in large omental WAT positively correlates with basal lipolysis. Furthermore, in participants matched for BMI, it is also positively correlated with apparent insulin sensitivity [22]. In this study, bioinformatics-based analysis revealed that *CIDEA* was significantly downregulated in the WAT of patients with T2DM and obesity. It was also involved in the oxidative phosphorylation, adipogenesis, and fatty acid metabolism pathways. Further validation using patient samples showed that both the mRNA and protein expression levels of CIDEA were significantly downregulated in the intra-abdominal large omental WAT of the T2DM+OB group. In contrast, they were significantly upregulated in the OB and NC groups. This suggests that *CIDEA* might have a protective role in T2DM and could serve as a biomarker for T2DM, as well as a potential therapeutic target. While our findings align with those of previous studies, the role of *CIDEA* in the development and progression of T2DM and the specific mechanisms involved warrant more in-depth analysis. *FSCN1* emerged as one of the key DEGs identified in this study. Its protein product, FSCN1, is a member of the fascin family of actin-binding proteins. This family promotes various patterns of cell migration, motility, adhesion, and intercellular interactions and is involved in the formation and stabilization of multiple cell protrusions [23]. Moreover, FSCN1 is highly expressed in several types of tumors and plays roles in biological processes like tumor cell migration, invasion, and metastasis. While FSCN1 has been extensively reported in the context of tumor development, no studies have yet detailed its specific role or mechanism in T2DM [24]. In this study, bioinformatics analyses indicated that *FSCN1* was notably upregulated in the WAT of patients with T2DM and obesity. It was involved in inflammation-related pathways, including the Wnt/β-linked protein, C3, interleukin-2, and transcriptional activator 5 signaling pathways. Moreover, the experimental validation results showed that the mRNA and protein expression levels of FSCN1 were significantly upregulated in the T2DM+OB group and significantly downregulated in the OB and NC groups in intra-abdominal large omental WAT. This aligned with the findings from the bioinformatics analysis. FSCN1 may regulate the expression of certain inflammatory factors and activate inflammatory signaling molecules and pathways [25, 26]. The deposition and expansion of WAT can stimulate the production of numerous immune cells and inflammatory cytokines, leading to IR in the organism. Its elevated expression aligns with the pathological characteristics of obesity accompanied by T2DM. Consequently, this study indicates that *FSCN1* is pivotal in predicting the risk of T2DM. It likely facilitates the onset of T2DM in patients with obesity by engaging in inflammatory pathways. Thus, *FSCN1* holds potential as a diagnostic biomarker and a therapeutic target for T2DM prevention and treatment.

## Conclusion

This study identified *CIDEA* and *FSCN1* as key DEGs in intra-abdominal large omental WAT in T2DM patients with obesity and those with simple obesity at the molecular, tissue, human, and gene levels. To our knowledge, this marks the first time *CIDEA* and *FSCN1* have been reported as potential predictive risk biomarkers and novel therapeutic targets for T2DM. Although the key genes identified in this study demonstrate specificity, we are missing data from clinical specimens and experimental samples for T2DM subgroups with a normal BMI. Consequently, subsequent studies should focus on expanded sample validation to delve deeper into the roles of *CIDEA* and *FSCN1* in T2DM pathogenesis.

## Supplemental Data

**Table S1:** Datasets of the expression profiles of three genes and the related information of samples

**Data set number**	**Sample type and number**		**Chip platform**	**Tissue source**	**Use**
	**Simple obesity**	**T2DM combined with obesity**			
GSE16415	5	5	GPL2986 ABI Human Genome Survey Microarray Version 2	Abdominal greater omentum WAT	Screening DEGs
GSE71416	6	14	GPL570 [HG-U133_Plus_2] Affymetrix Human Genome U133 Plus 2.0 Array	Abdominal greater omentum WAT	Screening DEGs
GSE29231	12	12	GPL6947 Illumina HumanHT-12 V3.0 expression beadchip	Abdominal greater omentum WAT	Validation of key DEGs

T2DM: Type 2 diabetes mellitus; WAT: White adipose tissue; DEGs: Differentially expressed genes.

**Table S2:** Primer information

**Gene**	**F**	**R**
*CIDEA*	5’-CCAGCACGTCCCCACTTG-3’	5’-CGTTAAGGCAGCCGATGAAG-3’
*FSCN1*	5’-CTGCTACTTTGACATCGAGTGG-3’	5’-GGGCGGTTGATGAGCTTCA-3’
β-actin	5’-CATCCGTAAAGACCTCTATGCCAAC-3’	5’-ATGGAGCCACCGATCCACA-3’

**Table S3:** List of differentially expressed genes (DEGs)

**Differentially expressed genes**	**Gene ID**
Upregulated genes	*fscn1, ifitm3, hcst, ifitm2, akr1a1, tmsb10, fcgbp, gabarap, aif1, s100a11, cd14, hla-drb4, ifitm1, tyrobp, ninj1, hla-dqb1, lilrb2, gpsm3, ptpn6, CYBA, CNDP2, CD63, RPS4X, CAPZB, TCIRG1, GNAI2, PYCARD, CLIC1, TMED3, HSPB1, NNMT, GMFG, C1QB, PLD3, YWHAH, RBP7, ARHGDIB, PDGFRB, PLA2G7, C11orf31*
Downregulated genes	*bche, cidea, tkt, cgn, aox1, echdc3, bnc1, gabarapl1, anxa3, nell2, rerg, fgf2, kctd3, rprm, msln, cldn1, c19orf33, adh1a, c6, sema3c, rnase4, tfpi2 SLC25A1, ADH1B, MAOA, F3, ADH1C, GHR, HAS1, KRT18, PRKAR2B, SLC25A3, PRG4, TOB1, EIF4EBP2, NDUFB5, SULF1, PSMC6, ME1, GHITM, TXNIP, PDHA1, CPE, MPDZ AADAC, CIDEC, CIRBP, PPP1CC, SDC4, TM4SF1, PODXL, ECHS1, KRT8, ACSL1, ST13, TMEM14B, RAD21, SPRY2, GNAI1, LRPPRC, ACLY, DLD, PGRMC1, IDH1, PFKFB3, OGN. cebpa, slpi, scd, ptprf, plscr4, cpa3, rpl6, apt5b, nrip1, sscp2, nipsnap3a, glul, nfia, cav2, azgp1, aldh2, cxcl6, sucl2, adipoq, uppk3b, itln1. ALDH1A1, GAS1, ID1*

**Table S4:** Differentially expressed genes (DEGs) screened by Lasso regression

**Gene**	**logFC**	**AveExpr**	**t**	***P***	**adj. *P***	**B**
*CIDEA*	−3.22814	7.94670	−4.13713	0.00027	0.22811	0.27090
*TKT*	−2.14666	8.48327	−4.03715	0.00035	0.24438	0.05083
*CGN*	−1.07246	3.55245	−4.00654	0.00038	0.24438	−0.01638
*FSCN1*	1.76968	9.07141	3.89069	0.00053	0.26688	−0.26994
*ECHDC3*	−3.77431	10.06490	−3.82827	0.00062	0.26688	−0.40596
*GABARAPL1*	−1.06818	6.60442	−3.76192	0.00075	0.26688	−0.55001
*ANXA3*	−1.43995	6.22773	−3.65673	0.00099	0.26688	−0.77711
*IFITM3*	8.46716	23.86306	3.59541	0.00117	0.26805	−0.90871
*RPRM*	−1.00709	4.32291	−3.46568	0.00164	0.27794	−1.18498
*HLA-DQB1*	1.51862	4.81935	2.67010	0.01221	0.43873	−2.78763

Adj.: Adjusted.

**Table S5:** Differentially expressed genes (DEGs) screened by the Boruta algorithm

**Gene**	**logFC**	**AveExpr**	**t**	***P***	**adj. *P***	**B**
*BCHE*	−1.51639	5.88721	−4.30341	0.00017	0.21939	0.63834
*CIDEA*	−3.22814	7.94670	−4.13713	0.00027	0.22811	0.27090
*TKT*	−2.14666	8.48327	−4.03715	0.00035	0.24438	0.05083
*CGN*	−1.07246	3.55245	−4.00654	0.00038	0.24438	−0.01638
*FSCN1*	1.76968	9.07141	3.89069	0.00053	0.26688	−0.26994
*ECHDC3*	−3.77431	10.06490	−3.82827	0.00062	0.26688	−0.40596
*BNC1*	−1.91808	4.05204	−3.81547	0.00065	0.26688	−0.43380
*ANXA3*	−1.43995	6.22773	−3.65673	0.00099	0.26688	−0.77711
*KCTD3*	−1.08268	5.26523	−3.46911	0.00163	0.27794	−1.17772
*C19orf33*	−1.03748	4.49285	−3.13507	0.00387	0.35756	−1.87282
*FCGBP*	1.09781	3.96257	3.05427	0.00475	0.38915	−2.03671
*ADH1C*	−18.32394	28.59561	−2.89339	0.00710	0.41460	−2.35725
*S100A11*	5.77296	18.75785	2.87479	0.00743	0.41640	−2.39377
*PSMC6*	−1.10777	8.13286	−2.66024	0.01250	0.44505	−2.80621
*MPDZ*	−1.15052	6.19494	−2.63068	0.01342	0.45038	−2.86168
*CNDP2*	1.16454	5.60392	2.54060	0.01658	0.46793	−3.02853
*GNAI2*	1.65070	9.13936	2.46037	0.01997	0.49172	−3.17424
*TMEM14B*	−1.10872	9.93471	−2.43570	0.02113	0.49727	−3.21848
*RBP7*	1.70303	10.76148	2.14044	0.04072	0.55085	−3.72544

Adj: Adjusted.
